# Water and electrolyte abnormalities in novel pharmacological agents for kidney disease and cancer

**DOI:** 10.1007/s10157-025-02635-6

**Published:** 2025-02-12

**Authors:** Maho Terashita, Masahiko Yazawa, Naoka Murakami, Akira Nishiyama, Electrolyte Winter Seminar Collaborative Group, Takuya Fujimaru, Yoshiro Fujita, Kazuhito Hirose, Kyogo Kawada, Toshiaki Monkawa, Masahiko Nagahama, Masatomo Ogata, Akihiro Ryuge, Yugo Shibagaki, Hideaki Shimizu, Hirofumi Sumi, Naoto Tominaga

**Affiliations:** 1 Center for Transplantation Sciences, Massachusetts General Hospital/Harvard Medical School, 55 Fruit Street, Boston, MA 02114 USA; 2 Division of Nephrology and Hypertension, Department of Internal Medicine, St. Marianna University School of Medicine, 2-16-1 Sugao, Miyamae-Ku, Kawasaki, Kanagawa 216-8511 Japan; 3 Division of Nephrology, Washington University in St. Louis, 1 Brookings Drive, St. Louis, MO 63130 USA; 4 Department of Pharmacology, Faculty of Medicine, Kagawa University, 1750-1 Ikenobe, Miki-Chou, Kida-Gun, Kagawa, 761-0793 Japan; 5 Department of Nephrology, St. Luke’s International Hospital, Tokyo, Japan; 6 Department of Rheumatology and Nephrology, Chubu Rosai Hospital, Nagoya, Japan; 7 Department of General Medicine, Tsukuba Medical Center Hospital, Tsukuba, Japan; 8 Department of Nephrology and Renal Replacement, Daido Hospital, Nagoya, Japan; 9 Medical Education Center, Keio University School of Medicine, Tokyo, Japan; 10 Ryuge Naika Otai Clinic, Naogya, Japan; 11 Division of Nephrology and Hypertension, Kawasaki Municipal Tama Hospital, Kawasaki, Japan

**Keywords:** Water and electrolyte disturbances, Immune checkpoint inhibitors, ICI, Sodium–glucose cotransporter-2 inhibitor, SGLT2 inhibitor, Non-steroidal mineral corticoid receptor antagonist, MRA, Hyponatremia, Hyperkalemia

## Abstract

This review article series on water and electrolyte disorders is based on the ‘Electrolyte Winter Seminar’ held annually for young nephrologists in Japan. This is the third article in this series that focuses on water and electrolyte disturbances caused by novel pharmacological agents for kidney disease and cancer. The advent of novel pharmacological agents in cardiorenal medicine and oncology has introduced both therapeutic benefits and challenges in managing medication-induced water and electrolyte disturbances. These medications, including sodium–glucose cotransporter-2 (SGLT2) inhibitors, non-steroidal mineralocorticoid receptor antagonists (ns-MRAs), and immune checkpoint inhibitors (ICIs), significantly impact water and electrolyte homeostasis. SGLT2 inhibitors used widely in diabetes mellitus, heart failure, and chronic kidney disease mitigate hyperkalemia and hypomagnesemia but increase the risk of hypernatremia in patients on fluid restriction. Conversely, they are beneficial for managing hyponatremia in the syndrome of inappropriate antidiuresis (SIAD). ns-MRAs, prescribed for diabetic kidney disease, exhibit a high risk of hyperkalemia, particularly when combined with renin–angiotensin system inhibitors. ICIs, a breakthrough in oncology, frequently induce hyponatremia through immune-related adverse events, such as hypophysitis and non-immune-related adverse events like SIAD. Understanding the pathophysiology of these disturbances and implementing timely interventions, including hormone replacement and water and electrolyte management, is critical for optimizing treatment outcomes.

## Introduction

This article, the third review by the Electrolyte Winter Seminar Collaborative Group, focuses on water and electrolyte disturbances caused by novel pharmacological agents for kidney disease and cancer, highlighting their benefits and adverse effects. Sodium–glucose cotransporter-2 (SGLT2) inhibitors are widely used not only for patients with diabetes mellitus (DM) but also for patients with congestive heart failure (CHF) and chronic kidney disease (CKD) regardless of DM status. SGLT2 inhibitors mitigate hyperkalemia and hypomagnesemia, commonly observed in patients with DM, CHF, or CKD. Hypernatremia is a potential risk for individuals on fluid restriction when using SGLT2 inhibitors due to increased renal free-water clearance. However, this effect has been utilized in randomized controlled trials (RCTs) investigating hyponatremia in patients with the syndrome of inappropriate antidiuresis (SIAD). Non-steroidal mineralocorticoid receptor antagonists (ns-MRAs) are widely prescribed for diabetic kidney disease (DKD) in clinical practice. Hyperkalemia remains a significant concern with MRAs, including ns-MRAs, particularly in patients with CHF, CKD, DM, or those receiving renin–angiotensin system (RAS) inhibitors as these groups are typically vulnerable to hyperkalemia. This review also explores the relationships between medications for patients with CKD, DM, and CHF, including angiotensin receptor/neprilysin inhibitors, endothelin-A receptor inhibitors, glucagon-like peptide-1 receptor agonists, and water and electrolyte disturbances. Immune checkpoint inhibitors (ICIs), a cancer therapy breakthrough, frequently induce hyponatremia (30–60%) through immune-related adverse events (irAEs) such as hypophysitis, SIAD, or hemodynamic disturbances. Proper diagnosis and tailored treatment based on the underlying causes, including fluid restriction or hormone replacement, facilitate the continuation of ICI therapy. This review mainly summarizes the pathophysiology of water and electrolyte disturbances associated with these innovative medications. Table 1 summarizes electrolyte disturbances in patients treated with novel pharmacological agents for kidney disease and cancer, categorized as commonly observed, clinically concerning, or beneficial effects without harm.

**Table 1:** Electrolyte disturbances in patients treated with novel pharmacological agents for kidney disease and cancer: commonly observed, clinically concerning, and beneficial effect

	Sodium (Na)		Potassium (K)		Calcium (Ca)		Phosphorus (P)		Magnesium (Mg)	
	Hypo-Na	Hyper-Na	Hypo-K	Hyper-K	Hypo-Ca	Hyper-Ca	Hypo-P	Hyper-P	Hypo-Mg	Hyper-Mg
Immune checkpoint inhibitor	⦾		✓		✓	✓	✓			
SGLT2 inhibitor	§	✓		§					§	
ARNI	✓			⦾						
ns-MRA	✓			⦾						
ERA										
GLP1-Ra			✓							

⦾: Commonly observed, ✓: Clinically concerning, §: Beneficial effect

SGLT2 inhibitor, sodium–glucose cotransporter-2 inhibitor; ARNI, angiotensin receptor–neprilysin inhibitor; ns-MRA, non-steroidal mineralocorticoid receptor antagonist; ERA, endothelin-1 receptor antagonist; GLP-1RA, glucagon-like peptide-1 receptor agonist

### Electrolyte disturbances associated with SGLT2 inhibitors

SGLT2 inhibitors are antihyperglycemic agents used in diabetes management. These agents block glucose and sodium reabsorption in the kidneys, promote renal glucose and sodium/chloride excretion, and stimulate tubuloglomerular feedback [1, 2]. Owing to their unique mechanism of blocking renal tubular channels, SGLT2 inhibitors exert diverse effects beyond reducing blood glucose levels. Clinical studies have demonstrated that SGLT2 inhibitors improve cardiac [3, 4] and renal outcomes [5] in diabetic and non-diabetic conditions, including CKD [6, 7] and heart failure [8]. Furthermore, SGLT2 inhibitors maintain optimal water and electrolyte balance and generally do not cause significant physiological electrolyte imbalances. However, the most serious electrolyte disturbance is hypernatremia, particularly in individuals on fluid restriction, as SGLT2 inhibitors cause osmotic diuresis, called renal free-water clearance, via glycosuria.

#### Potassium

Initially, concerns on hyperkalemia emerged with the introduction of SGLT2 inhibitors into clinical practice [3], as patients with DM are more susceptible to hyperkalemia, specifically in the presence of renal dysfunction. Furthermore, the initial decrease in glomerular filtration rate (GFR), namely “initial dip or initial drop,” observed soon after the initiation of SGLT2 inhibitors, also supports this concern [9]. Nevertheless, a meta-analysis found no significant increase in serum potassium levels or hyperkalemia events with SGLT2 inhibitors compared with placebo [10]. Rather, SGLT2 inhibitors were associated with a lower risk of hyperkalemia, particularly in patients with diabetic nephropathy who concurrently received RAS inhibitors or MRAs [11].

Multiple mechanisms have been proposed for how SGLT2 inhibitors affect potassium homeostasis. Urinary potassium excretion can be affected by SGLT2 inhibitors. Urinary potassium excretion is determined by potassium secretion in the renal distal tubule and collecting duct. Several factors stimulate renal potassium excretion, including aldosterone, high distal sodium delivery, high urine flow rate, high potassium in tubular cells, negative charge in the collecting duct lumen, and metabolic alkalosis. Several mechanisms may reduce hyperkalemia risk in patients using SGLT2 inhibitors. Palmer et al. thoroughly reviewed potassium regulation by SGLT2 inhibitors [12]. They summarized opposing mechanisms affecting renal potassium excretion, either increasing or limiting it. First, SGLT2 inhibitors promote distal sodium delivery by inhibiting sodium reabsorption in the proximal tubules. Second, increased urinary pH in the distal tubules arises from the inhibition of sodium–hydrogen exchanger 3 (NHE3), located in the proximal tubules and interlinked with SGLT2. Onishi et al. reported that empagliflozin increased renal sodium excretion in wild-type mice but not in NHE3-KO mice [13], indicating that the increase in distal sodium delivery observed with SGLT2 inhibitors was due to NHE3 inhibition rather than SGLT2 inhibition. NHE3 inhibition also increases urinary pH, a key driver of increased renal potassium excretion in the distal nephrons. Third, SGLT2 inhibitors may transiently increase aldosterone levels shortly after initiation [14]. However, the effect of aldosterone on renal potassium excretion may be limited because increased sodium delivery to the macula densa by SGLT2 can inhibit the release of renin, followed by aldosterone release [12, 15, 16]. Fourth, SGLT2 inhibition increases glucosuria that raises osmolality in the tubular lumen, resulting in osmotic diuresis and a high urine flow rate. Fifth, increased glucagon levels may also play a role in maintaining lower serum potassium levels. However, the effect of glucagon on serum potassium levels is much smaller than that of insulin or aldosterone [12].

Conversely, the risk of hypokalemia does not appear to be substantial in clinical trials [8]. Decreased renin release is one possible mechanism that limits renal potassium excretion. However, the effect of SGLT2 inhibitors on the renin–angiotensin–aldosterone system remains controversial. Increased sodium delivery to the macula densa by SGLT2 inhibitors inhibits renin release. Conversely, SGLT2 inhibitor-induced reduced blood volume and decreased blood pressure can stimulate renin release. In a meta-analysis of clinical studies involving patients with type 2 DM (T2DM), plasma renin activity remained significantly higher in patients using SGLT2 inhibitors for ≤ 3 months [17]. Other possible mechanisms include increased activity of the thiazide-sensitive sodium chloride cotransporter on the distal convoluted tubule by allosteric modification of calcium-sensing receptor (CaSR), which is significantly activated by glucosuria induced by SGLT2 inhibition [18]. Additionally, increased delivery of α-ketoglutaric acid to the distal nephron leads to inhibition of epithelial sodium channel (ENaC) in the collecting ducts [12].

Despite initial concerns regarding the potential of SGLT2 inhibitors to induce hyperkalemia, accumulating evidence from clinical studies has demonstrated otherwise. These findings underscore the therapeutic advantages of SGLT2 inhibitors in patients at high risk of hyperkalemia, including those with DM and CKD and those using RAS inhibitors or MRAs. Although these medications exhibit opposing effects on renal potassium excretion in renal tubules, they are unlikely to cause hypokalemia. SGLT2 inhibitors reduce the risk of hyperkalemia, making them user-friendly options from the perspective of potassium management.

### Water–sodium balance

#### Hypernatremia

Case reports suggest SGLT2 inhibitors may be associated with hypernatremia [19, 20]. Hypernatremia results from osmotic diuresis caused by glucosuria, which induces renal free-water excretion through urine. Hypernatremia is rarely observed in patients who can drink water freely or respond to thirst. All patients with hypernatremia in the aforementioned case reports were unable to drink freely. The authors concluded that clinicians should consider discontinuing SGLT2 inhibitors when patients are hospitalized, ill, or undergoing surgery as they may drink less and retain a more concentrated solution than urine. Kitada et al. demonstrated the renoprotective effects of SGLT2 inhibitors in the context of water conservation [21]. SGLT2 inhibitors initially promote osmotic diuresis, which subsequently activates the body’s water conservation system through urea accumulation in the renal medulla. This mechanism facilitates urea-driven renal free-water reabsorption [21]. Consequently, SGLT2 inhibitors alone are unlikely to cause hypernatremia. Physicians should pay close attention to this preventable electrolyte abnormality, particularly in patients on “Sick Day” or those unable to drink water freely.

### SGLT2 inhibitors as a treatment option for hyponatremia

SGLT2 inhibitors can be used to treat hyponatremia. SGLT2 inhibitors may become a new treatment option for hyponatremia, specifically SIAD, due to osmotic diuresis and increased free-water clearance. Notably, empagliflozin significantly increased serum sodium levels in hospitalized patients or outpatients with hyponatremia due to SIAD in two RCTs comparing empagliflozin with placebo [22, 23]. The medication was well-tolerated, with no reported hypoglycemia in the empagliflozin group. However, a few patients in the empagliflozin group showed transient renal dysfunction and returned to hyponatremia after discontinuation. Fluid restriction may need to be relaxed in patients with SIAD treated with SGLT2 inhibitors to avoid a decrease in GFR. The treatment duration in these studies was only 4 days for hospitalized patients and 28 days for outpatients, and whether SGLT2 inhibitors should be continued to prevent the recurrence of hyponatremia due to SIAD remained unclear. In an outpatient study, 79% (11 of 14) of the patients showed persistent hyponatremia at the follow-up visit after medication discontinuation [23]. Furthermore, the two aforementioned RCTs used a 25 mg dose of empagliflozin. In Japan, a 25 mg dose of empagliflozin is approved for the treatment of T2DM but not for CKD or CHF. No data exist on the relationship between SGLT2 inhibitor dosage and its effect on hyponatremia. Moreover, longitudinal data on the use of SGLT2 inhibitors for SIAD remains lacking, and no country have approved them for SIAD.

#### Magnesium

Hypomagnesemia is common in patients receiving SGLT2 inhibitors (i.e., those with DM, CKD, and CHF), with its incidence being 25% in T2DM [24], 14.7% in CKD [25], and 19–37% in heart failure [26]. Hypomagnesemia is associated with an increased risk of cardiovascular outcomes, CKD progression [27], and all-cause mortality [28].

SGLT2 inhibitors increase serum magnesium levels. A meta-analysis of 25 RCTs involving patients with T2DM reported a significant association between SGLT2 inhibitors and increased serum magnesium by 0.07 mmol/L (0.17 mg/dL) [29]. SGLT2 inhibitors also increased serum magnesium levels in Japanese patients with CKD (median eGFR, 33.5 mL/min/1.73 m^2^), specifically in patients with hypomagnesemia at baseline [30]. Possible mechanisms by which SGLT2 inhibitors increase serum magnesium levels have been reviewed. SGLT2 inhibitors enhance serum magnesium levels through mechanisms affecting the kidney, bone, and gastrointestinal systems. In the kidney, they increase paracellular magnesium reabsorption in the thick ascending limb via elevated transmembrane potential gradients and enhance active transcellular reabsorption in the distal convoluted tubules by upregulating the transient receptor potential cation channel subfamily M member 6 (TRPM6) channels, influenced by factors, such as increased epidermal growth factor, glucagon, and reduced oxidative stress. SGLT2 inhibitors may enhance magnesium absorption via active transcellular transport mediated by TRPM6/7 channels in the gastrointestinal tract. Additionally, systemic effects, such as reduced circulating insulin levels, may promote magnesium mobilization from the bone and intracellular stores into the extracellular compartment, further contributing to improved magnesium homeostasis [24].

A small case series has demonstrated the effectiveness of SGLT2 inhibitors in managing refractory hypomagnesemia in patients with renal magnesium wasting [31]. The study attributed the improvement in hypomagnesemia to a reduction in fractional excretion of magnesium (FEMg) as the underlying mechanism [31]. Notably, the drug effects of SGLT inhibitors (selective inhibitors of SGLT2 or dual inhibitors of SGLT1 and SGLT2) may play a role in magnesium homeostasis. Sotagliflozin, a dual inhibitor of SGLT1 and SGLT2, exerts a greater glycosuric effect and enhances glucagon secretion. Additionally, sotagliflozin increases serum magnesium levels compared to dapagliflozin, a selective SGLT2 inhibitor, while not reducing FEMg [32]. This effect may be mediated by increased intestinal magnesium absorption through SGLT1 in the gastrointestinal tract [32]. Sotagliflozin is not currently available in Japan. Furthermore, whether hypomagnesemia correction using SGLT2 inhibitors improves cardiovascular and renal outcomes in a large RCT [33] remains unclear.

### Self-assessment question 1

**Case 1:** A 65-year-old woman receiving dapagliflozin 5 mg, metformin 500 mg, and semaglutide 3 mg for T2DM; pravastatin 10 mg for dyslipidemia; prednisolone 5 mg and montelukast sodium 10 mg for bronchial asthma; and vonoprazan 10 mg for chronic gastritis was hospitalized with subarachnoid hemorrhage and underwent coil embolization. All her original medications were resumed on day 2, regardless of appetite or food consumption. An isotonic sodium chloride solution was administered to prevent cerebral vasospasms. She did not eat well and developed a fever due to pneumonia. On day 5, the patient developed dysarthria and dysphagia. Laboratory reports on day 9 revealed 170 mEq/L serum sodium, 133 mg/dL blood glucose, 61 mEq/L urine sodium, 19.9 mEq/L urine potassium, and 518 mOsm/kg urine osmolality, with urine glucose over 1000 mg/dL.

Which of the following medications or conditions is MOST related to hypernatremia?

A. Arginine vasopressin deficiency (formerly known as central diabetes insipidus).

B. Arginine vasopressin resistance (formerly known as renal diabetes insipidus).

C. Dapagliflozin.

D. Metformin.

E. Prednisolone.

The correct answer is C.

SGLT2 inhibitors increase renal free-water excretion due to osmotic diuresis by increasing glucose excretion through urine. Under normal circumstances, this is not a problem because thirst promotes drinking behavior, but when patients cannot drink freely and appropriately or when a hypertonic (even isotonic saline) solution is administered, as in this case, the risk of hypernatremia is high. In such patients, temporary suspension of SGLT2 inhibitor administration and monitoring of serum sodium levels, urine volume, and urinary electrolytes to check renal free-water clearance are necessary. Arginine vasopressin deficiency or resistance is also associated with low urine osmolality. Metformin and steroids are not associated with hypernatremia. This case is based on a CEN case report published in 2024 [19].

### Electrolyte disturbances related to ns-MRAs

The mineralocorticoid receptor (MR) is critical in regulating fluid, electrolyte, and hemodynamic homeostasis. In the context of cardiorenal diseases, particularly in patients with CKD, the pathological overactivation of the MR has been confirmed [34, 35]. This overactivation of MR is associated with the promotion of inflammation and fibrosis, ultimately contributing to the progression of end-organ damage, such as kidney or heart failure. Consequently, MRAs are considered promising therapeutic options to attenuate pro-inflammatory and pro-fibrotic pathways, enhancing cardiorenal protection.

#### Hyperkalemia

MRAs can elevate serum potassium levels by blocking the MR, the most important site of potassium handling in nephrons. Furthermore, patients taking MRAs are typically vulnerable to hyperkalemia due to concomitant medications, such as RAS inhibitors, heart failure, CKD, and DM. Hyperkalemia is a significant factor that limits tolerance to MRAs [36].

Finerenone, a non-steroidal and selective MRA, has emerged as a promising alternative to traditional steroidal MRAs such as spironolactone or eplerenone, offering minimal unfavorable effects and potential advantages in treating cardiorenal diseases. Two major clinical trials have been published regarding the use of finerenone in patients with CKD and T2DM: FIDELIO-DKD [37] and FIGARO-DKD [38], involving adults with T2DM and CKD (eGFR > 25 or > 30 mL/min/1.73 m^2^ and albuminemia) treated with a maximum-tolerated labeled dose of RAS inhibitors. The primary composite outcomes included kidney failure, a sustained decrease of at least 40% in eGFR or death from renal causes (FIDELIIO-DKD), and a composite of death from cardiovascular causes, nonfatal myocardial infarction, nonfatal stroke, or hospitalization for heart failure (FIGARO-DKD). Treatment with finerenone improved the primary composite outcomes in both trials (17.8% vs. 21.1%; hazard ratio, 0.82; *p* = 0.001 in FIDELIO-DKD; 12.4% vs. 14.2%; hazard ratio, 0.87; *p* = 0.03 in FIGARO-DKD). The finerenone group exhibited more hyperkalemia events than the placebo group in both trials, particularly in patients with low eGFR [39]. The risk of hyperkalemia with finerenone is considered low compared to that with spironolactone, a classical steroidal MRA. In the ARTS study [40], finerenone (BAY 94–8862, 10 mg maximum) was compared with open-label spironolactone (25–50 mg). Briefly, the inclusion criteria were adults with heart failure with reduced ejection fraction and eGFR of 30–60 mL/min/1.73 m^2^. In this study, 94.9% of the patients took RAS inhibitors. Finerenone was associated with significantly smaller mean increases in serum potassium level (0.04–0.30 vs. 0.45 mEq/L) and lower incidences of hyperkalemia (5.3 vs. 12.7%) than spironolactone [40]. In addition, a comparative post hoc analysis was reported using finerenone from the FIDELITY trials, combined data from the FIDELIO-DKD and FIGARO-DKD trials, and spironolactone data from the AMBER trial [41, 42]. Among these common patients for nephrologists (with eGFR approximately 36–37 mL/min/1.73 m^2^, T2DM, and treatment-resistant hypertension), hyperkalemia (> 5.5 mEq/L) occurred in 12% of the patients in the finerenone group compared to 64% in the spironolactone group. Discontinuation rates owing to high potassium levels were lower in the finerenone group (0.3% vs. 23%).

Several factors contribute to the lower hyperkalemia risk with finerenone use. First, the half-life of finerenone is 2–3 h in healthy individuals [43]. Similar results have been reported in patients with CKD [44]. Finerenone metabolites lack pharmacological activity. Finerenone has a shorter half-life compared to spironolactone, whose active metabolites persist for over 12 h in healthy individuals and even longer in patients with CKD or heart failure [45, 46]. Second, finerenone is equally distributed between the heart and kidneys (1:1) in rats [47], whereas spironolactone favors kidney distribution. This difference in tissue distribution may explain the lower hyperkalemia frequency and favorable effects of finerenone on the heart.

Table 2 shows the incidence of hyperkalemia with steroidal MRAs (spironolactone/eplerenone) and ns-MRAs (finerenone) from landmark randomized trials. In an aging population, increased finerenone use in patients with CKD, low renal function, or multiple comorbidities potentially elevates hyperkalemia risk. Following the RALES trial [48], spironolactone was widely prescribed, leading to an increase in hyperkalemia-related adverse effects [49]. Further real-world evidence is required in this regard.

**Table 2:** Incidence of hyperkalemia following treatment with steroidal- and non-steroidal mineral corticoid antagonists according to the large randomized controlled trials

	MRA	Target population	eGFR (mL/min/1.73 m^2^)	Baseline potassium (mEq/L)	Diabetes (%)	EF (%)	RAS inhibitors (%)	Loop diuretics (%)	SGLT2 inhibitor (%)	Hyperkalemia > 5.5 mEq/L (placebo)	Hyperkalemia > 6.0 mEq/L (placebo)
FIDERIO-DKD	ns-MRA Finerenone	DKD	44.3 ± 12.6	4.37 ± 0.46	100	N/A	99.9	56.6*	4.6	21.7% (9.8%)	4.5% (1.4%)
FIGARO-DKD	ns-MRA Finerenone	DKD	67.8 ± 21.7	4.33 ± 0.43	100	N/A	99.9	47.6*	8.4	13.5% (6.4%)	2.3% (1.2%)
FINEARTS-HF	ns-MRA Finerenone	EF > 40%, heart failure	61.9 ± 19.4	4.4 ± 0.5	40.5	52.6 ± 7.8	79.5	87.2	13.1	14.3% (6.9%)	3.0% (1.4%)
EMPHASIS-HF	s-MRA Eplerenone	EF < 30%, heart failure	71.2 ± 21.9	4.3 ± 0.4	33.7	26.2 ± 4.6	94.0	84.3*	0	11.8% (7.2%)	2.5% (1.9%)
RALES	s-MRA Spironolactone	NYHA III/IV	N/A	N/A	N/A	25.6 ± 6.7	95.0	100	0	N/A	2% (1%)
Ref. 41	ns-MRA Finerenone s-MRA Spironolactone	DKD CKD with resistant hypertension	35–37	4.6–4.7	100% in FIDELITY 50% in AMBER	N/A	99–100	98–100^a^	N/A	Finerenone: 11.6% Placebo: 3.3% Spironolactone: 64.2%	N/A

N/A, not applicable; ns-MRA; non-steroidal mineral corticoid receptor antagonist; s-MRA, steroidal mineral corticoid receptor antagonist; eGFR, estimated glomerular filtration rate; RAS, renin–angiotensin inhibitor; SGLT, sodium–glucose cotransporter; DKD, diabetic kidney disease; EF, ejection fraction; NYHA, New York heart association

^a^Defined as just “diuretics”

### Concomitant use of MRAs with SGLT2 inhibitors

SGLT2 inhibitors show promise in preventing hyperkalemia associated with MRA use. Guidelines strongly recommend SGLT2 inhibitors and MRAs as part of the “Fantastic Four” for heart failure and reduced ejection fraction (HFrEF) [50] or the “Four Pillars” for DKD with albuminuria [51]. Recent studies emphasize the continuous use of RAS inhibitors [52] or SGLT2 inhibitors [53] to enhance medication efficacy against CVD and CKD progression. The concomitant use of SGLT2 inhibitors and RAS inhibitors/MRAs should be encouraged to ensure continued cardiorenal protection, particularly in patients with hyperkalemia. In the FIDERIO-DKD trial, co-administration of SGLT2 inhibitors reduced hyperkalemia incidence (8.1 vs. 18.7%) in patients receiving MRA [54].

#### Hyponatremia

MRAs rarely cause hyponatremia. The FIDELITY analysis of the FIDELIO-DKD and FIGARO-DKD trials showed hyponatremia prevalence of 1.2% and 0.8% in the finerenone (eGFR < 60 mL/min/1.73 m^2^) and placebo groups, respectively [55]. A few patients discontinued finerenone because of hyponatremia. In the EPHESUS study with eplerenone, hyponatremia incidence (serum sodium level < 135 mEq/L) was higher in the eplerenone group (15% vs. 11%, *p* = 0.0001) [56]. Hyponatremia due to MRAs likely results from increased renal sodium wasting due to ENaC inhibition and impaired urinary dilution [57, 58].

### Electrolyte disturbances related to angiotensin receptor/neprilysin inhibitor (ARNI)

ARNI comprises a combination of sacubitril, a neprilysin inhibitor, and valsartan, an angiotensin II type 1 receptor blocker (ARB). Sacubitril inhibits neprilysin, increasing vasoactive peptide concentrations, reducing vasoconstriction, and promoting diuresis. ARNI significantly lowers mortality and hospitalization risk in patients with heart failure compared to ACE inhibitors or ARBs.

### Hyperkalemia

ARNI may increase serum potassium levels, similar to ACE inhibitors or ARBs, due to RAS inhibition. A meta-analysis found no difference in hyperkalemia incidence between ARNI and ACE inhibitor/ARB groups [59]. However, some studies indicate that hyperkalemia associated with ARNI is less frequent or milder compared to ACE inhibitors or ARBs [60, 61]. This outcome is partly attributed to its lower risk of worsening renal function despite its blood pressure-lowering effects. ARNI also demonstrated a lower incidence of hyperkalemia compared to enalapril in patients with HFrEF receiving MRAs, primarily spironolactone [62]. Additionally, serum potassium levels did not increase with ARNI in patients with end-stage kidney disease undergoing dialysis [63]. Although hyperkalemia remains a limitation of RAS inhibitors, ARNI represents a safer option for high-risk patients. Recent heart failure management strategies, including ARNI, MRA, and ACE-I/ARB, accommodate higher serum potassium levels (i.e., < 6.5 mEq/L). Instead of discontinuing RAS inhibitors, potassium binders are increasingly used to maintain therapeutic doses in HF [64].

### Hyponatremia

ARNI can rarely cause hyponatremia. Several case reports describe both acute and chronic onset hyponatremia in patients with heart failure receiving ARNI treatment [65, 66]. Whether ARNI significantly increases the incidence of hyponatremia remains unclear, as heart failure alone can also cause this condition. One proposed mechanism for ARNI-induced hyponatremia involves the accumulation of endogenous natriuretic peptides due to neprilysin inhibition, which leads to enhanced natriuresis and subsequent volume depletion [65].

### Electrolyte disturbances related to endothelin-A receptor antagonists (ERAs) and glucagon-like peptide-1 receptor agonists (GLP-1RAs)

#### ERAs

ERAs may emerge as agents for kidney diseases, particularly IgA nephritis and diabetic kidney disease. Clinical investigations have found no significant electrolyte disorders. A notable side effect of ERAs is fluid retention, a key concern in clinical use [67]. A post hoc analysis of the SONOR study suggests that this side effect may be mitigated by combining an SGLT2 inhibitor, which synergistically reduces albuminuria [68].

#### GLP-1RAs

GLP-1RAs may reduce major adverse cardiovascular events and improve renal function in patients with T2DM and kidney diseases [69, 70]. Like SGLT2 inhibitors, GLP-1 receptor agonists are associated with a lower incidence of hyperkalemia than DPP-4 inhibitors [71]. This effect is likely due to increased sodium and water delivery to the distal nephron and elevated urinary pH from NHE3 inhibition in the proximal tubule, promoting renal potassium excretion [72, 73]. This benefit may enable the continuation of RAS inhibitors or MRAs in patients at risk of hyperkalemia.

### Electrolyte disturbances related to immune checkpoint inhibitors (ICIs)

ICIs enhance host immune cell ability to identify and eliminate cancer cells [74]. Immune checkpoints are molecules on immune cells, such as T cells, that regulate immune responses. They maintain self-tolerance and prevent autoimmunity. ICIs enhance the ability of the immune system to recognize and kill tumor cells. They block inhibitory signals, such as cytotoxic T lymphocyte-associated antigen-4 (CTLA4), enabling T cells to target cancer cells. ICIs include monoclonal antibodies targeting CTLA4, programmed cell death receptor-1 (PD-1), and PD-1 ligand (PD-L1). These agents exhibit favorable responses and are widely used for various solid tumors, including melanoma, non-small-cell lung cancer, and clear-cell renal cancer. With increased clinical use, the effects of ICIs on electrolytes have been increasingly recognized. ICIs increase immune system activity, leading to a unique spectrum of autoimmune and inflammatory disorders known as irAEs. Electrolyte abnormalities in patients receiving ICIs include frequent issues, including hyponatremia, hypokalemia, and hypercalcemia [75]. Table 3 summarizes the mechanism of each electorate disturbance in patients with cancer using ICIs.

**Table 3:** Mechanism of each electorate disturbance in patients with cancer using immune checkpoint inhibitors

	Mechanism
Hyponatremia	ICIs specific causes: Primary- or secondary- adrenal deficiency (hypophysitis, isolated ACTH deficiency, adrenalitis) and hypothyroidism (hypophysitis, thyroiditis) Common causes in cancer patients: SIAD (volume depletion, nausea/vomiting) and hemodynamic disturbance
Hypokalemia	Renal tubular acidosis and colitis
Hypercalcemia	Hypothyroidism, hyperthyroidism, primary or secondary adrenal deficiency, sarcoid-like reaction, PTHrP secretion
Hypocalcemia	CaSR auto-antibody, and hypoparathyroidism
Hyperphosphatemia	Tumor lysis syndrome and hypoparathyroidism
Hypophosphatemia	Renal tubular acidosis and Fanconi syndrome

CaSR, calcium-sensing receptor; ICIs, immune checkpoint inhibitors; PTHrP, parathyroid hormone-related protein; SIAD, syndrome of inappropriate antidiuresis

#### Hyponatremia

Hyponatremia is the most common water–electrolyte disturbance in patients with cancers, even without ICIs. ICIs are associated with increased the incidence of hyponatremia compared to cytotoxic chemotherapy [76]. Hyponatremia frequency in patients with ICIs is higher in real-world settings (approximately 30–60%) than in clinical trials [75, 76]. Immune-related hypophysitis, a well-known irAE, is a unique cause of hyponatremia in patients receiving ICIs. Hyponatremia can be the first symptom prompting an irAEs diagnosis [77]. Hypophysitis can cause panhypopituitarism or adrenocorticotropic hormone (ACTH)-isolated deficiency. Cortisol inhibits the production and release of arginine vasopressin (AVP). AVP production and release can increase during cortisol deficiency [78]. Clinical studies report immune-related hypophysitis incidence between 0.5% and 9%. Hypophysitis can occur in patients treated with ICIs, with incidence varying by regimen. Garon-Czmil et al. reported the use of ipilimumab (CTLA-4 inhibitor) monotherapy or combination therapy in most hypophysitis cases, with over 50% of cases with grade 3 severity (125–129 mEq/L with symptoms or 120–124 mEq/L regardless of symptoms) and most (90%) with corticotropic deficiency. Notably, onset times varied with ipilimumab (79 [14–506] days), being significantly shorter than other ICIs (approximately 150 days) [79]. Adrenalitis, another irAE, can cause adrenal deficiency and hyponatremia, though less frequently. Hypothyroidism, resulting from immune-related hypophysitis or thyroiditis, is a common irAE. Although hypothyroidism can cause hyponatremia, it rarely leads to severe cases [80]. Patients with hyponatremia receiving ICIs should undergo a comprehensive workup to determine its causes.

Causes of hyponatremia in the setting of ICI use vary, and not all ICI-related cases are due to irAEs. Seethapathy et al. found that common causes of grade 3 or 4 hyponatremia were SIAD (35%) and hemodynamic disturbance (20%) [76]. Endocrinopathies accounted for only 7%. It is important to investigate the underlying causes of hyponatremia rather than assuming immune-related hypophysitis in ICI-treated patients [76]. This study identified risk factors for severe hyponatremia (< 125 mEq/L) in patients receiving ICIs. CTLA-4 inhibitors and diuretics are significant risk factors [76].

ICI-related hyponatremia should be treated according to its underlying causes. SIAD treatment in patients treated with ICIs is the same as in the general population [78]. These treatments include fluid restriction, oral urea or hyperosmotic agents, and vasopressin antagonists (vaptans). In the case of endocrinopathies, such as hypophysitis, as irAEs, long-term hormone replacement is usually required, as these conditions are considered irreversible. ICIs are usually tolerated when hormone replacement is initiated and maintained [78].

#### Hypokalemia

Hypokalemia is a common electrolyte abnormality in patients receiving ICIs. The incidence of hypokalemia is 17–24% in the Food and Drug Administration (FDA) database [75]. ICIs, specifically anti-PD-1 therapy, have been implicated in a novel form of renal tubular acidosis (RTA) with hypokalemia. This condition is associated with non-gap metabolic acidosis, impaired renal ammonia excretion, hypokalemia, preserved urinary acidification, and normal proximal tubule citrate transport. PD-L1 expression in renal proximal tubules suggests that anti-PD-L1 therapy may induce vacuolization and disrupt ammoniagenesis. The FDA database reports a higher incidence of hypokalemia with PD-L1 inhibitors compared to other ICIs [75]. These findings highlight a unique mechanism of ICI-associated RTA, termed “type V RTA [81].”

### Hypercalcemia and hypocalcemia

Hypercalcemia, an electrolyte disorder associated with malignancy, should not be overlooked. Before the introduction of ICIs, hypercalcemia of malignancy (HCM), primarily caused by cancer, has been well known. The most frequent cause of HCM is parathyroid hormone-related protein (PTHrP) secretion from tumor cells, responsible for approximately 70% of cases. Local osteolytic hypercalcemia from skeletal metastasis accounts for 20% of cases. In the context of ICIs, ICI-induced hypercalcemia must be considered in the differential diagnosis [82]. The mechanisms of ICI-associated hypercalcemia involve immune-mediated and tumor-related pathways. First, endocrinopathies play significant roles in hyperthyroidism, hypothyroidism, and adrenal insufficiency. Hyperthyroidism contributes to PTH-independent hypercalcemia through increased bone resorption and calcium mobilization, whereas primary adrenal insufficiency enhances calcium reabsorption and bone release, potentially mediated by altered 1-alpha hydroxylase activity. Second, sarcoid-like granulomas, known to occur with ICIs, contribute to hypercalcemia through 1-alpha hydroxylase activity. This enzyme converts inactive vitamin D to its active form, increasing intestinal calcium absorption and elevating serum calcium levels. Third, HCM may occur in patients receiving ICI therapy. Increased secretion of PTHrP can stimulate calcium resorption from bones and renal reabsorption, further contributing to hypercalcemia. Finally, hyperprogression or pseudoprogression, which is observed in some patients during ICI therapy, can exacerbate hypercalcemia [82]. These mechanisms underscore the complex interplay among ICIs, immune system activation, and calcium homeostasis, requiring careful monitoring and management of hypercalcemia in patients undergoing ICI therapy.

Hypocalcemia is relatively rare, affecting approximately 5% of patients. One report attributed this high incidence to non-albumin correction [83]. The incidence of hypocalcemia after ICI therapy may be lower than reported in some clinical trials [82]. This electrolyte abnormality is also affected by immune-related autoantibodies against parathyroid glands, especially CaSR-activating antibodies [84].

### Self-assessment question 2

**Case 2:** A 74-year-old male with advanced melanoma treated with ipilimumab and nivolumab experienced general fatigue and nausea after the fourth nivolumab cycle. A routine lab test showed serum Na 125 mEq//L, serum osmolality 265 mOsm/kg H_2_O, creatinine 0.9 mg/dL, BUN 20 mg/dL, blood glucose 112 mg/dL, urine Na 112 mEq/L, and urine osmolality 362 mOsm/kg H_2_O.

Which ONE of the following is the MOST appropriate next step?

A. Check serum cortisol.

B. Check serum free T3 and T4.

C. Get MRI scan of pituitary gland.

D. Prescribe hydrocortisone.

E. Promptly Stop the ICI.

The correct answer is A.

This case presented hypotonic hyponatremia in a patient receiving ICI. Elevated urine osmolality and sodium levels despite low serum osmolality suggest secondary or primary adrenal insufficiency due to an irAE or SIAD. Hypothyroidism rarely causes symptomatic hyponatremia. Checking for low cortisol levels is recommended for diagnosis.

## Conclusion

Novel medications in fields, such as cardiorenal medicine and oncology, have introduced challenges in managing drug-induced electrolyte disturbances. These innovative medications, widely used for therapeutic advancement, impact water and electrolyte homeostasis. Water and electrolyte imbalances can lead to severe complications if undiagnosed or mismanaged, potentially affecting outcomes and requiring the cessation of beneficial treatments. Given their broad use, nephrologists should understand the monitoring and management of water and electrolyte disorders associated with these medications. Regular laboratory testing, awareness of high-risk populations, and prompt correction of imbalances are essential to prevent complications. Early recognition and intervention can enable continued treatment while mitigating risks.

## Data Availability

Not applicable.
